# Viral RNA in Mosquitoes (Diptera: Culicidae) Collected between 2019 and 2021 in Germany

**DOI:** 10.3390/v15122298

**Published:** 2023-11-23

**Authors:** Janine Rau, Katharina Köchling, Mandy Schäfer, Birke A. Tews, Claudia Wylezich, Günter A. Schaub, Doreen Werner, Helge Kampen

**Affiliations:** 1 Friedrich-Loeffler-Institut, Federal Research Institute for Animal Health, Südufer 10, 17493 Greifswald, Germany; mandy.schaefer@fli.de (M.S.); birke.tews@fli.de (B.A.T.); claudia.wylezich@fli.de (C.W.); helge.kampen@fli.de (H.K.); 2 Zoology/Parasitology Department, Ruhr-University, Universitätsstr. 150, 44801 Bochum, Germany; guenter.schaub@rub.de; 3 Leibniz Centre for Agricultural Landscape Research, Eberswalder Str. 84, 15374 Müncheberg, Germany; doreen.werner@zalf.de

**Keywords:** mosquito-borne viruses, Usutu virus, Sindbis virus, West Nile virus, Batai virus, *Culex pipiens* complex

## Abstract

Due to globalisation and climate change, mosquito-borne pathogens are emerging in new areas on all continents, including Europe, which has recently faced outbreaks of dengue, chikungunya and West Nile fever. The present study complements previous investigations to evaluate the circulation of mosquito-borne viruses in Germany, with the aim of identifying potential vector species and risk areas. Mosquitoes collected from 2019 to 2021 and identified to species or species group level were screened for viruses of the families Flaviviridae, Peribunyaviridae and the genus *Alphavirus* of the family Togaviridae. In total, 22,528 mosquitoes were examined, thus providing the most comprehensive study on West Nile virus (WNV) circulation so far in the German mosquito population. Usutu virus (USUV) RNA was detected in six samples, Sindbis virus (SINV) RNA in 21 samples and WNV RNA in 11 samples. Samples containing RNA of USUV and WNV consisted of mosquitoes collected in the East German federal states of Brandenburg, Saxony and Saxony-Anhalt, while samples with RNA of SINV originated from more widespread locations. Although minimum infection rates have remained relatively low, the intensity of virus circulation appears to be increasing compared to previous studies. Continuous mosquito screening contributes to the early detection of the introduction and spread of mosquito-borne pathogens.

## 1. Introduction

After the disappearance of malaria in the mid-20th century in Europe [1], mosquitoes as vectors of disease agents have been largely ignored for a considerable period of time in Germany. Apparently, no mosquito-borne pathogens of serious health risk to humans had been circulating for decades. However, several of the at least 52 mosquito species presently known to occur in Germany [2,3] have been shown to be vector-competent for one pathogen or the other. These include native species, such as *Culex pipiens* and *Aedes vexans*, and invasive species, such as *Ae. albopictus* and *Ae. japonicus* [4].

In addition to mosquito species newly emerging in Europe, globalisation and climate change have facilitated mosquito-borne pathogens to spread and establish in regions in which they did not occur before [5]. Thus, the number of mosquito-borne disease outbreaks have recently been increasing in southern Europe. For example, in 2007, the first epidemic of chikungunya fever occurred in northern Italy, followed by further outbreaks in 2010, 2014 and 2017 in France and Italy [6]. Autochthonous dengue cases were observed in several countries in southern Europe (Croatia, France, Italy and Spain) from 2010 onwards [7], including a large epidemic in Portuguese Madeira in 2012 [8]. With few exceptions, West Nile fever (WNF), mainly caused by West Nile virus (WNV) lineage 1, was registered only sporadically in Europe until the mid-1990s but generally became more frequent and aggressive with the emergence of WNV lineage 2 in the early 21st century [9,10]. From 2011 to 2022, human outbreaks were reported in several European countries, including Greece, Croatia, Serbia, Hungary, Italy and Germany [11]. These events culminated in the large WNF epidemic in 2018, which affected considerable parts of southern and Central Europe [12]. Notably, 2018 was also the first year in which WNV was detected in Germany [13].

After the disappearance of autochthonous malaria and several decades without noteworthy transmission of mosquito-borne pathogens [14], several mosquito-borne viruses had previously been detected in mosquitoes collected in Germany. These include the *Ťahyňa* virus (TAHV) [15], Sindbis virus (SINV) [16,17,18], Batai virus (BATV) [17], Usutu virus (USUV) [17,19] and WNV [20,21]. Some of those are known to be able to infect humans where they can cause mild flu-like symptoms [22,23]. Birds act as reservoirs, and the viruses are thought to be transmitted by a variety of mosquito species, including those of the *Cx. pipiens* complex, which are widely distributed in Germany [22]. SINV occurs primarily in northern Europe, where it recurrently leads to epidemics of Sindbis fever in late summer [24,25], whereas TAHV is widespread in continental Europe [26,27,28].

Human cases with USUV are extremely rare, mainly affect immunocompromised persons, are commonly associated with fever and rash, and can involve the central nervous system [29,30]. USUV emerged in 2001 in Austria, where it caused mass mortality among black birds [31]. It was first detected in Germany in 2010 in a mosquito pool collected in the southwestern part of the country [19]. One year later, USUV led to a massive die-off among the blackbird population in the same region [32]. While USUV became endemic in southwestern Germany it was only sporadically detected in other parts of Germany [33,34,35] until 2016 when numerous dead blackbirds tested positive also in the federal states of North Rhine-Westphalia, Saxony and Saxony-Anhalt [36,37]. The virus continued to spread, and in 2018, it was the cause of mass mortality among blackbird populations throughout the country [38].

WNV usually circulates between wild birds and mosquitoes, but it can also be transmitted to other vertebrates [39]. Some groups of birds, horses and humans are susceptible and may develop disease symptoms [10,40]. WNV is highly life-threatening to horses and can cause WNF or West Nile neuroinvasive disease in humans, although most infections remain asymptomatic or present with mild flu-like symptoms [40]. Both horses and humans constitute dead-end hosts, which cannot pass on the virus to mosquitoes [40,41]. In 2018, the first infections with WNV were diagnosed in Germany in birds and horses [13], while the first mosquito-borne human cases were observed in 2019, and the first human fatal case was registered in 2020 [42]. Subsequently, WNV-RNA was found in native mosquitoes in the *Culex pipiens* complex [20,21].

The present study follows up on a study by Scheuch et al. [17]. It provides further data on the spatiotemporal circulation of viruses in mosquitoes collected in Germany, facilitating conclusions on suspected vector species and contributing to transmission risk assessments and the design of mosquito and mosquito-borne disease management plans.

## 2. Materials and Methods

### 2.1. Mosquito Collection

**Figure 1:**
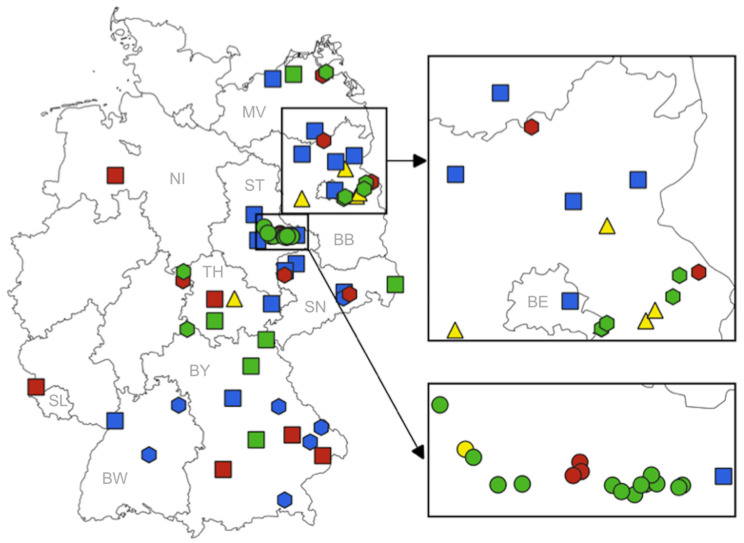
Trap locations in 2019, 2020, and 2021. Colours represent the years of sampling (yellow—2019, green—2020, red—2021, blue—2020 and 2021). Different trap types are marked by different symbols (square—BG-Sentinel trap, dot—EVS trap, triangle—BG-Sentinel UV-light trap, hexagon—popup bag). Federal states: BB—Brandenburg, BE—Berlin, BW—Baden-Wuerttemberg, BY—Bavaria, MV—Mecklenburg-Western Pomerania, NI—Lower Saxony, SL—Saarland, SN—Saxony, ST—Saxony-Anhalt, TH—Thuringia.

Mosquitoes were collected from April to October (occasionally in November) 2019 to 2021 in the framework of the German mosquito monitoring programme and associated research projects. More than 50 sites were sampled by using different methods with a focus on eastern Germany (Figure 1). This focus was made for logistical reasons, but is backed by recent studies on birds and horses that indicated virus circulation particularly in East German regions [43,44]. Trap-site selection criteria included local support for trap attendance and specific features such as high variety of available hosts in zoos, human proximity in private gardens, and previous detection of mosquito-borne viruses in the Elbe River floodplains in Saxony-Anhalt.

In 2020 and 2021, garden popup bags were deployed without attractants in private gardens to serve as resting places for mosquitoes during blood digestion [45]. They were sampled with an electric aspirator once a week. In addition to 2020 and 2021, attractant traps such as BG-Sentinel traps (Biogents, Regensburg, Germany) and encephalitis virus surveillance (EVS) traps (BioQuip Products, Compton, CA, USA) were used to collect host-seeking mosquitoes. The BG-Sentinel traps were operated for 24 h once a week either in bird/animal parks, in a peatland or in private gardens using CO_2_ from gas tanks and BG-Lure (Biogents) as attractants. Occasionally, additional mosquito samples were collected from the area surrounding the trapping sites and close-by concealed spaces in some of the bird/animal parks using an aspirator. The EVS traps, equipped with dry ice as a CO_2_ source, were operated three times overnight in July, August and September, in 2020 and 2021, respectively, in the floodplains of the Elbe River in the federal state of Saxony-Anhalt. Mosquitoes collected as by-catch in a biting midge monitoring project in 2019, in which BG-Sentinel UV-light traps (Biogents) were used, were also examined. The biting midge traps were run once a week for 24 h on farms with animal husbandry or in animal/wildlife parks.

All mosquitoes collected by CO_2_-baited traps or an aspirator were killed and preserved by placing them in a deep freezer (−20 °C) in the morning after a trapping night or after finalizing the aspirating activities at each location, respectively. Mosquitoes captured as by-catch in the biting midge study were conserved in 70% ethanol.

### 2.2. Mosquito Identification

Mosquitoes were kept deep-frozen or in alcohol until nucleic acid extraction in the laboratory. Prior to extraction, morphological identification to species or complex/group level was done following the determination key by Becker et al. [46] on a chilling table or in a dish with 70% ethanol, respectively, using a stereomicroscope.

*Anopheles maculipennis* complex females were processed individually for nucleic acid extraction. All further mosquito females were pooled according to species/species group, collection site and collection date, with up to 16 specimens in the case of smaller species, such as those belonging to the genera *Culex* and *Aedes*, and with up to five specimens in the case of larger species, such as those belonging to the genus *Culiseta*.

Mosquitoes were homogenised in 500 µL (single mosquitoes) or 750 µL (pools) serum-free ZB5d medium (FLI-intern cell culture medium = Eagle’s minimal essential medium with Earle’s and Hank’s salts plus non-essential amino acids) [47] and the addition of 1 µL (single mosquitoes) or 1.5 µL (pools) of a ready-to-use gentamicin-amphotericin mixture and 5 µL (single mosquitoes) or 7.5 µL (pools) of a ready-to-use penicillin-streptomycin mixture (ThermoFisher Scientific, Dreieich, Germany) by adding three stainless steel beads with a diameter of 3 mm (DIN 5401 G40 material 1.4034; Martin, Gauting, Germany) and agitating the samples for 2 min at 30 Hz in a TissueLyser II (Qiagen, Hilden, Germany). The homogenate was shortly centrifuged (3400× *g*), and 200 µL of supernatant was used for simultaneous DNA and RNA extraction using the NucleoMag VET kit (Macherey-Nagel, Düren, Germany) according to the manufacturer’s instructions.

For genetic identification, *An. maculipennis* complex specimens were subjected to a conventional polymerase chain reaction (PCR) assay targeting the internal transcribed spacer 2 (ITS2) region [48,49]. Mosquito pools containing viral RNA and consisting of *Cx. pipiens* complex specimens were retrospectively identified genetically according to species or biotype using a multiplex real-time PCR assay [50]. Mosquitoes that were morphologically indistinguishable due to the absence of unique characters were genetically identified by amplifying and sequencing the cytochrome oxidase c subunit I (COI) gene [51,52]. Specimens belonging to species known to be reliably identifiable neither morphologically nor genetically in the female stage were evaluated as a species group (e.g., *Cs. morsitans*/*fumipennis*).

### 2.3. Virus RNA Screening

Mosquito females collected in 2019 and early 2020 were screened for viruses of the families Flaviviridae and Peribunyaviridae and of the genus *Alphavirus* of the family Togaviridae using the QuantiTect SYBR Green RT-qPCR kit (Qiagen) with primers designed by Vina-Rodriguez et al. [53], Lambert and Lanciotti [54] and Eshoo et al. [55] (Table 1). Thermoprofile and high-resolution melting-curve analyses were applied as described by Vina-Rodriguez et al. [53]. Each RT-real-time PCR was performed with respective positive controls (Flaviviridae: WNV; Peribunyaviridae: Bunyamwera orthobunyavirus, Oropouche virus, TAHV and *Wyeomyia* orthobunyavirus; *Alphavirus*: chikungunya virus) and nuclease-free water as a negative control. If the melting curve analysis gave a positive result, the PCR product was purified by gel electrophoresis (1.5% agarose), extracted by means of the QIAquick Gel Extraction kit (Qiagen) and sequenced with the forward primer to confirm virus identity by sequence comparison with GenBank (https://blast.ncbi.nlm.nih.gov/Blast.cgi; accessed on 12 October 2023) [21].

Since WNV and USUV had been the only mosquito-borne Flaviviridae ever detected in Germany and other Flaviviridae were not found in the mosquitoes collected in 2019 and most of 2020, the costly and time-consuming Pan-Flaviviridae-PCR was replaced by a multiplex RT-qPCR capable of differentiating WNV lineage 1, WNV lineage 2 and USUV [56] in late 2020. Accordingly, mosquitoes collected in late 2020 and in 2021 were only screened for these viruses/lineages, but no other Flaviviridae, each PCR being performed with positive controls of WNV lineages 1 and 2 and USUV (supplied by C. Körsten, Friedrich-Loeffler-Institut, Greifswald, Germany) and nuclease-free water as a negative control. In addition, the similarly costly and time-consuming RT-real-time PCR used to detect Peribunyaviridae was no longer performed on mosquito collections from late 2020 and 2021 since the screening of samples from 2019 and most of 2020 never yielded positive results.

To ensure that BATV was not missed due to methodological problems after having negatively tested thousands of mosquitoes, the BATV detection system was re-assessed in early 2021 using diluted test samples prepared from a mosquito extract containing 1.33 × 10^8^ virus particles (supplied by K. Franzke, Friedrich-Loeffler-Institut). Since titres of BATV as low as 0.133 TCID_50_/mL could be detected by the PCR assay, suggesting that the results had been correctly negative, screening for Peribunyaviridae was discontinued due to the unsuccessful, time-consuming and costly procedure. All RT-real-time PCRs were run on a CFX96 Real-Time System (BioRad, Munich, Germany).

An annual minimum infection rate (MIR) ([number of positive pools/total specimens tested] × 1000) was determined for each collection site, assuming that in cases of amplicon production only one mosquito specimen per pool contained virus RNA, independent of the size of the pool [57].

## 3. Results

A total of 22,528 mosquitoes were examined (2657 pools and 5107 single specimens). The mosquitoes could be assigned to 30 species or species groups/complexes. A total of 573 mosquitoes (corresponding to 85 pools and 56 single specimens) consisting of five species/species groups were analysed from the 2019 collections, 9400 mosquitoes (1190 pools and 4236 single specimens) with 30 species/species groups from the 2020 collections, and 12,555 mosquitoes (1382 pools and 815 single specimens) with 29 species/species groups from the 2021 collections.

Thirty-eight single specimens or pools of mosquitoes contained RNA of SINV, USUV or WNV lineage 2, with the majority of the detections (63.2%) being observed in *Cx. pipiens* complex mosquitoes. The RNA of WNV lineage 1 was not detected. Each viral sequence generated could be unambiguously assigned to a single virus species with a similarity of more than 96% with GenBank database entries. Sequences believed to be mosquito-specific viruses were not included.

**Figure 2:**
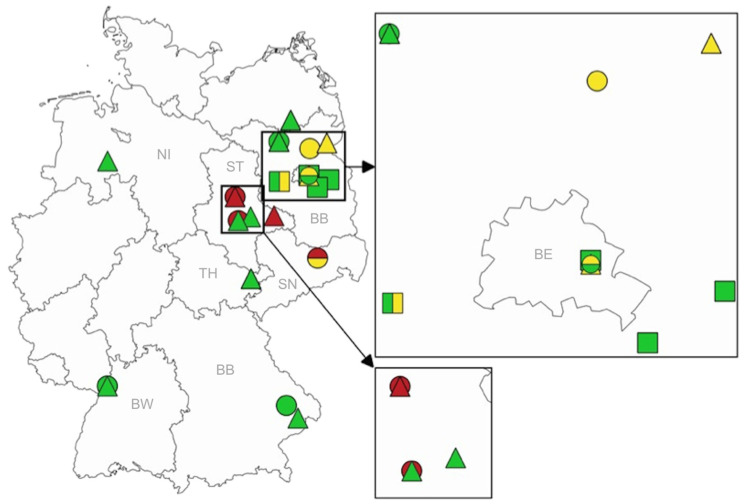
Collection sites of mosquitoes in Germany tested positive for viruses from 2019 to 2021 (green—SINV-RNA, red—WNV-RNA, yellow—USUV-RNA, square—2019, dot—2020, triangle—2021). Federal states: BB—Brandenburg, BE—Berlin, BW—Baden-Wuerttemberg, BY—Bavaria, NI—Lower Saxony, SN—Saxony, ST—Saxony-Anhalt, TH—Thuringia.

In 2019, six out of 141 mosquito samples (4.3%, MIR 10.5) contained RNA of SINV (Table 2, Supplementary Table S1). The mosquitoes were collected from April to October in Berlin, Beerfelde, Groß Kreutz and Dannenreich (Figure 2, Supplementary Table S1). One mosquito containing SINV-RNA could be assigned to the species *An. messeae* (Groß Kreutz), one mosquito to *An. maculipennis* s.s. (Berlin), three mosquitoes to *Cx. pipiens* biotype *pipiens* (Berlin, Beerfelde, Dannenreich) and one mosquito to *Cx. torrentium* (Berlin). Furthermore, one pool from the Groß Kreutz sampling site contained RNA of USUV in September 2019 (Table 2, Supplementary Table S1). It consisted of a mixture of *Cx. pipiens* biotypes *pipiens* and *molestus* or hybrids of them. Thus, in 2019, 0.7% (MIR 1.7) of the samples signalled the presence of USUV-RNA (Figure 2, Supplementary Table S1), but WNV-RNA could not be detected.

In 2020, SINV-RNA was detected in 5 out of 5426 samples (0.1%, MIR 0.5) (Table 2, Supplementary Table S1). The mosquitoes were collected from May to September at the locations Kunsterspring, Moos, Eggenstein-Leopoldshafen and Berlin (Figure 2). The mosquito species/species groups included *Ae. sticticus* (one pool, one single mosquito, Moos), *Ae. annulipes* group (one single mosquito, Kunsterspring), *An. daciae* (one single mosquito, Eggenstein-Leopoldshafen) and *Cx. pipiens* biotype *pipiens* (one single mosquito, Berlin). In addition to SINV-RNA, USUV-RNA was detected in three samples in 2020 (0.1%, MIR 0.3) (Table 2, Supplementary Table S1). The mosquitoes were caught in August and September in Berlin, Schorfheide and Dresden. Samples were composed of *Ae. vexans* (one single mosquito, Schorfheide), *Cx. pipiens* biotype *pipiens* (one single mosquito, Dresden) and *Cx. pipiens* biotypes *pipiens* and *molestus* or their hybrids (one pool, Berlin) (Figure 2, Supplementary Table S1). WNV-RNA was detected in three single mosquitoes (0.1%, MIR 0.3) in 2020 (Table 2, Supplementary Table S1). The mosquitoes were collected between June and September in Magdeburg, Dresden and Bernburg. All three mosquitoes were *Cx. pipiens* biotype *pipiens*.

In 2021, all three viruses were found again (Table 2, Supplementary Table S1). Ten out of 2197 samples (0.5%, MIR 0.8) were positive for SINV-RNA. The mosquitoes in these samples had been collected from June to November at the locations Kunsterspring, Eggenstein-Leopoldshafen, Gera, Neustrelitz, Irgenöd, Goldenstedt, Bernburg and Aken. The RNA-containing samples included *An. claviger* (single mosquito, Kunsterspring), *Ae. cinereus/geminus* (one pool, Eggenstein-Leopoldshafen), *Ae. sticticus* (one pool, Eggenstein-Leopoldshafen), *Ae. vexans* (one pool, Neustrelitz), *Cs. morsistans/fumipennis* (one pool; Goldenstedt), *Cx. modestus* (one pool, Aken), *Cx. pipiens* biotype *pipiens* (single mosquito, Gera; one pool, Goldenstedt), and not-identifiable taxa of the *Cx. pipiens* complex (2 pools, Bernburg and Irgenöd). In two samples (0.1%, MIR 0.2), USUV-RNA was demonstrated in 2021: a single *Ae. vexans* (Angermünde, in June) and a mixture of *Cx. pipiens* biotype *pipiens* and *Cx. torrentium* (pool from Berlin, in August) (Figure 2, Supplementary Table S1). Finally, eight pools (0.4%, MIR 0.6) contained RNA of WNV in 2021. The mosquitoes in seven samples had been caught at the sampling site Magdeburg and of one sample in Wittenberg between June and August. The mosquitoes in three samples were identified as *Cx. pipiens* biotype *pipiens* (one pool and one single mosquito from Magdeburg, one pool from Wittenberg), while five pools from Magdeburg could not be specified further than *Cx. pipiens* complex.

MIRs ranged from 0.4 to 36.1 (Table 2), with Berlin showing the highest MIR for RNA of SINV in 2019 (36.1). The sampling sites Kunsterspring and Neustrelitz displayed the highest MIRs for SINV-RNA in 2020 and 2021 (11.0 and 12.8, respectively). Regarding USUV-RNA, the highest MIR was observed in Groß Kreutz in 2019 (2.9), Schorfheide and Dresden in 2020 (14.9 and 14.7, respectively) and Berlin in 2021 (4.1). For RNA of WNV, Magdeburg recorded the highest MIR in 2020 with 15.2, closely followed by Dresden with 14.7. Magdeburg again exhibited the highest MIR for WNV-RNA (11.5) in 2021.

## 4. Discussion

Within three years of mosquito monitoring (2019–2021), 38 out of 7764 examined mosquito samples tested positive for virus RNA via PCR. Calculated MIRs exhibit variability across years, locations and viruses, ranging from 0.4 to 36.1. However, caution should be exercised when interpreting MIRs, as a small sample size may lead to artificially high MIR values resulting from incidental findings. In that regard, the MIR concept is less accurate than the infection prevalence concept, which represents the proportion of positive specimens to the total number of specimens examined in individual screening. Nevertheless, for reasons of time and costs, analysing a representative number of individual mosquitoes from a huge area with an anticipated low level of virus circulation is hardly feasible.

A major part (12 single specimens and nine pools) of the tested mosquito pools was found positive for SINV-RNA. In 2009, the first molecular survey for SINV in Germany demonstrated the occurrence of SINV strains in the southwestern part of the country [16]. Further studies showed sporadic occurrences of the virus in mosquitoes collected in central and northeastern Germany [17,18]. In addition, SINV has also been detected in resident birds in Germany [58,59]. The present study confirms the ongoing circulation of SINV by findings at 11 locations.

The detection was mostly limited to single RNA-containing samples at each location, indicating virus circulation but without much room for interpretation. However, at three locations, SINV-RNA was detected twice, with larger sample sizes resulting in lower MIR values that are likely to be representative of an actual low prevalence. Notably, SINV-RNA was detected in three single mosquitos (MIR 36.1) in 2019 in Berlin, suggesting a higher viral prevalence at that site compared to other sampling locations. This high MIR was not reproducible in 2020 and 2021, possibly due to differences in sampling locations within the city of Berlin in 2019.

While SINV-RNA was found in three different mosquito taxa (*Cx. torrentium, Cx. pipiens* and *An. maculipennis* s.l.) in 2009, it was demonstrated in 11 mosquito taxa in the present study. These include both taxa known to be vector-competent for SINV, such as *Ae. cinereus*, *Ae. sticticus*, *Cx. pipiens* biotype *pipiens* and *Cx. torrentium,* and taxa that have been found carrying the virus in the field [4,60]. The latter represent specimens of the *An. maculipennis* complex, within which SINV-RNA had been detected in Germany before, but without identification of the very mosquito species [16]. By contrast, species identification was conducted in the present study, and SINV-RNA was detected in *An. daciae* and *An. maculipennis* s.s. *Anopheles claviger*, which was also demonstrated to harbour SINV-RNA, had not previously been linked to this virus. However, apart from *Cx. pipiens* biotype *pipiens* and *Cx. torrentium*, none of these mosquito taxa are known to be specifically ornithophilic. Instead, they are rather considered to exhibit an indiscriminative feeding behaviour or a preference for mammals [46]. Moreover, *Ae. vexans*, in which SINV-RNA was detected, had been previously excluded as a vector of SINV based on laboratory studies [61]. The specimens containing virus-RNA were not visibly blood-fed but must have either been infected with subsequent virus replication or have ingested a virus-containing bloodmeal not too long ago for RNA remnants to be detectable. It has to be kept in mind that virus demonstration is not necessarily equal to vector competence [4].

Scheuch et al. [17] screened 97,648 mosquitoes, collected between 2011 and 2016, and found SINV-RNA in three out of 4144 mosquito pools, resulting in an MIR of 0.03. In the present study, three to five years later, fewer mosquitoes were screened but an MIR of 0.9, all years and locations included, was found for SINV-RNA. However, far smaller pools (with up to 16 individuals) were tested in the present study than in the study by Scheuch et al. [17] (up to 50 individuals). Unsurprisingly, when the pools are larger, more dilution of viral RNA takes place, resulting in a higher risk of missing viral RNA by PCR [62]. Furthermore, the chance of having more than one infected mosquito in a positive pool increases with the number of individuals per pool, although the MIR remains the same. Thus, a relatively high number of infected mosquitoes can hide in a low number of positive pools, taking effect particularly when the virus concentrates geographically, e.g., in hotspots.

Our study suggests that SINV has recently been spreading in the mosquito population in Germany. In addition, one of the *Cx. pipiens* complex pools, which had been collected by an aspirator in a hibernation site (cellar) in November 2021 in Bernburg and a single *Cx. torrentium* trapped very early in the season (mid-April) contained SINV-RNA, suggesting overwintering of the virus in hibernating *Culex* females. This finding is consistent with a study from Sweden, where several overwintering *Cx. pipiens* specimens were found positive for SINV-RNA [63].

In Finland and Sweden, SINV has been circulating for decades and is responsible for numerous human infections [24]. About every seven years, an outbreak of Pogosta disease, the regional name of SINV infection, occurs in the human population in Finland [25]. When comparing the MIRs for SINV-RNA of all examined mosquitoes in the present study (MIR 0.9) with MIRs from an endemic area in Sweden (MIR 16.7) [64], the MIRs in Germany turn out low. Moreover, no human cases of the disease have been reported from Germany so far. However, given the apparent spread of SINV in the country, there is a compelling need for further investigations. Additionally, raising awareness among healthcare workers about the potential risks associated with SINV is of significant importance.

USUV has been found in birds Germany-wide since 2018 [38], and the present study confirms its circulation in eastern Germany. The sampling locations with mosquito samples containing USUV-RNA were primarily in Berlin and Brandenburg, with the exception of one single mosquito found in Saxony. These areas had already been identified, among others, as potential risk areas for USUV [36], and multiple birds tested positive for USUV-RNA in those areas in 2019 and 2020 [65]. The observed MIR for USUV-RNA was generally low, ranging from 1.2 to 4.2. The single findings of USUV-RNA in Schorfheide, producing an MIR as high as 14.9, and in Dresden, producing an MIR of 14.7, cannot be considered representative due to the limited sample size. Nevertheless, even the detection of a single mosquito containing USUV-RNA indicates the persistent circulation of USUV in eastern Germany. Scheuch et al. [17] demonstrated USUV-RNA in only two out of 4144 mosquito pools (97,648 specimens) examined between 2011 and 2016 from all over the country, originating from already-known hotspot regions. In 2016, many blackbirds were found dead around the city of Leipzig (federal state of Saxony) and tested positive for USUV-RNA [37]; however, none of the mosquitoes collected by Scheuch et al. [17] during the same year around Leipzig contained RNA of this virus. These contrasting findings suggest that MIRs/infection prevalences observed in the mosquito population were probably not representative and demonstrate the importance of complementary monitoring of wild birds and mosquitoes for comprehensive surveillance.

The samples with USUV-RNA consisted of specimens of the *Cx. pipiens* complex or *Ae. vexans*, field-collected samples of which had already previously been found to contain USUV-RNA [4]. The predominant number of positive samples belonged to the *Cx. pipiens* complex (*n* = 4), which are recognised vectors of the virus [65,66]. Although two specimens of *Ae. vexans* tested positive for USUV-RNA, its vector competence in laboratory studies was relatively low [66], suggesting a minor epidemiological role.

In the present study, no WNV-RNA was detected in 2019, but four single mosquitoes and seven pools from four different locations in East Germany tested positive in 2020 and 2021. The detected RNA belonged to WNV lineage 2, the strain recently circulating in Germany, and all positive locations agree with previous findings of WNV in birds and horses [44]. All RNA-containing mosquitoes were collected in animal parks, with some of them having reported losses in their bird population due to WNF (U. Lender, Zoo Magdeburg, and T. Suckow, Tiergarten Bernburg, pers. comm.). Furthermore, studies on birds from zoos in those areas had demonstrated previous WNV infections [43]. Mosquitoes containing WNV-RNA, however, had not been detected before at those locations. The MIR varied from 3.1 to 15.2 in 2020 and from 0.4 to 11.6 in 2021. At most sites, only one or two mosquito samples contained viral RNA, but Magdeburg peaked with 8 WNV-RNA-positive out of 117 samples examined (6.8%; MIRs: 15.2 in 2020, 11.6 in 2021). Magdeburg is located in an area of high WNV activity, which includes the German federal states of Saxony, Saxony-Anhalt and Berlin and some regions of Brandenburg [67]. Our results agree with those findings and indicate an increased WNV infection risk in Magdeburg. In future studies, it would be interesting to investigate individual mosquitoes from Magdeburg to be able to determine the actual infection prevalence. Based on the pooling of the mosquitoes, it can so far only be concluded that at least eight but no more than 62 of the 672 mosquitoes tested contained WNV-RNA, equivalent to infection prevalences of 1.2 and 9.2%, respectively. It is nevertheless advisable to implement measures in the affected zoo to decimate the mosquito population, aiming at reducing the risk of WNV transmission to both zoo animals and human visitors.

In addition to demonstrating SINV and USUV, Scheuch et al. [17] found BATV to be the most common mosquito-borne virus in mosquitoes from Germany. Although the authors sampled similar regions, no BATV could be detected in the present study. This may partially be explained by the fact that species of the *An. maculipennis* complex, which are considered the main vectors of BATV in Europe [22], were caught only in low numbers. However, Scheuch et al. [17] also detected the virus in mosquito taxa other than the *An. maculipennis* complex such as *Ae. vexans* and specimens of the genera *Culex* and *Culiseta*, although these are unlikely to be vector-competent [4]. Eventually, the lack of detecting BATV in this study does not necessarily indicate that the virus was not present anymore in these regions of Germany.

The mosquito collections between 2019 and 2021 were done with different trapping methods and at various sites. Animal parks turned out to be especially productive locations for trapping due to the high abundance and variety of potential mosquito breeding habitats and hosts, particularly birds, and the willingness of their staff to practically support the study. The use of garden popup bags as resting places was expected to increase the yield of blood-fed mosquitoes as compared to attractant traps (up to 20% according to preliminary experiments by Sauer et al. [45]) and therefore promised a higher probability of pathogen detection. However, while the yield of blood-fed mosquitoes was in fact higher with popup bags, most of the samples containing viral RNA (63.2%) were detected among *Cx. pipiens* complex pools collected with BG-Sentinel traps (Supplementary Table S1). This could be due to the fact that specimens of the *Cx. pipiens* complex, which are commonly considered the main European vectors of SINV, USUV and WNV, are generally caught in higher numbers with BG-Sentinel traps than with popup bags [45,68]. For future studies, employing consistent trap types across sites is recommended to mitigate potential biases attributed to trapping effectiveness. 

## 5. Conclusions

By examining mosquitoes collected in Germany, the occurrence and distribution of mosquito-borne viruses were investigated using PCR in order to obtain infection prevalences/MIRs and assess potential transmission risks to humans and animals. While the MIRs found demonstrate increasing mosquito-borne virus circulation in space and time in Germany and may suggest mosquito species contributing to natural transmission cycles, they are not appropriate to deduce vector species or epidemiological roles of mosquito species without further information from vector competence studies. Certainly, positive mosquitoes must have imbibed virus (or viral RNA)-containing blood but, since complete mosquitoes were processed and examined by PCR, data do not provide information on whether viable virus was present, able to replicate and disseminate in the very mosquito species, and whether this mosquito would be able to transmit the virus.

Notwithstanding, this study provides comprehensive data on WNV circulation in the mosquito population for the first time in Germany. Pools found to contain WNV-RNA only consisted of taxa belonging to the *Cx. pipiens* complex, which are the accepted major vectors of WNV. Although the MIR was comparably high in Magdeburg, overall data are still scarce and too low to assess infection risks for animals and humans by themselves alone. MIRs of mosquitoes are, however, a helpful data source supplementing animal (bird and horse) infection prevalence for early warning purposes.

In order to detect the establishment of new mosquito-borne viruses at an early stage and to control the spread of mosquito-borne viruses in Germany, regular monitoring of the German mosquito fauna for such viruses is advisable. Certainly, such monitoring should be carried out nationwide.

## Figures and Tables

**Table 1:** Primers and probes used to detect RNA of the various families, groups, lineages or species of viruses (WNV—West Nile virus, USUV—Usutu virus), including controls.

Primer/Probe	Sequence	Amplicon Length [bp]	Target Region	Virus Target	Reference
PFlav-fAAR	5′-TACAACATGATGGGAAAGAGAGAGAARAA-3′	266	NS5	Flaviviridae	[53]
PFlav-rKR	5′-GTGTCCCAKCCRGCTGTGTCATC-3′				
WN-LCV-F1	5′-GTGATCCATGTAAGCCCTCAGAA-3′	76	3′-UTR	WNV line-ages 1 and 2	[56]
WN-LCV-R1	5′-GTCTGACATTGGGCTTTGAAGTTA-3′				
USU-F	5′-ACGGCCCAAGCGAACAGAC-3′	91	3′-UTR	USUV	
USU-R	5′-GGCTTGGGCCGCACCTAA-3′				
WN-LCV-S1	5′-FAM-AGGACCCCACATGTT-3′MGB	–		WNV lineage 1	
WN-LCV-S2	5′-VIC-AGGACCCCACGTGCT-3′MGB	–		WNV lineage 2	
USU-S	5′-CY5-CGAACTGTTCGTGGAAGG-3′BBQ	–		USUV	
Cal/Bwa forward	5′-GCAAATGGATTTGATCCTGATGCAG-3′	210	N ORF	Peribunya-viridae	[54]
Cal/Bwa reverse	5′-TTGTTCCTGTTTGCTGGAAAATGAT-3′				
Bun forward	5′-CTGCTAACACCAGCAGTACTTTTGAC-3′	250			
Bun reverse	5′-TGGAGGGTAAGACCATCGTCAGGAACTG-3′				
*Wyeomyia* forward	5′-ATGTCTGAAATTGTATTTGATGATATTGG-3′	230			
*Wyeomyia* reverse	5′-TATTTCGATTCCCCGGAAAGT-3′				
*Oropouche* forward	5′-GGCCCATGGTTGACCTTACTTT-3′	300			
*Oropouche* reverse	5′-ACCAAAGGGAAGAAAGTGAAT-3′				
VIR2052F	5′-TGGCGCTATGATGAAATCTGGAATGTT-3′	139	nsP4	*Alphavirus* (Togaviridae)	[55]
VIR2052R	5′-TACGATGTTGTCGTCGCCGATGAA-3′				

**Table 2:** Minimum annual infection rates according to year, site and virus (SINV—Sindbis virus).

Year	Location	Total no. of Tested Mosquitoes	No. of Tested Single Mosquitoes	No. of Tested Pools	Virus-RNA Found	Number of Positive Specimens/Pools	Minimum Infection Rate
2019	Beerfelde	30	12	9	SINV	1	33.3
	Berlin	83	24	14	SINV	3	36.1
	Dannenreich	75	4	13	SINV	1	13.3
	Groß Kreutz	340	4	60	SINV	1	2.9
	Groß Kreutz	340	4	60	USUV	1	2.9
2020	Berlin	238	26	25	SINV	1	4.2
	Eggenstein-Leopoldshafen	815	628	35	SINV	1	1.2
	Kunsterspring	91	25	12	SINV	1	11.0
	Moos	1318	763	111	SINV	2	1.5
	Berlin	238	26	25	USUV	1	4.2
	Dresden	68	11	20	USUV	1	14.7
	Schorfheide	67	25	13	USUV	1	14.9
	Bernburg	319	87	32	WNV	1	3.1
	Dresden	68	11	20	WNV	1	14.7
	Magdeburg	66	8	12	WNV	1	15.2
2021	Aken	388	18	47	SINV	1	2.6
	Bernburg	574	133	61	SINV	1	1.7
	Eggenstein-Leopoldshafen	837	66	90	SINV	2	2.4
	Gera	84	4	14	SINV	1	11.9
	Goldenstedt	1184	78	154	SINV	2	1.7
	Irgenöd	488	122	45	SINV	1	2.1
	Kunsterspring	131	22	22	SINV	1	7.6
	Neustrelitz	78	20	13	SINV	1	12.8
	Angermünde	839	43	111	USUV	1	1.2
	Berlin	245	23	34	USUV	1	4.1
	Magdeburg	606	21	76	WNV	7	11.6
	Wittenberg	2374	29	172	WNV	1	0.4

## Data Availability

Data supporting the conclusions of this article are included within the article and its Supplementary Tables.

## Supplementary Materials

The following supporting information can be downloaded at: https://www.mdpi.com/article/10.3390/v15122298/s1, Table S1: Virus detections in mosquitoes from Germany in 2019 to 2021.

## References

[1] Boualam M.A., Pradines B., Drancourt M., Barbieri R. (2021). Malaria in Europe: A historical perspective. Front. Med..

[2] Werner D., Kowalczyk S., Kampen W. (2020). Nine years of mosquito monitoring in Germany, 2011–2019, with an updated inventory of German culicid species. Parasitol. Res.

[3] Kuhlisch C. (2022). Discovery of *Aedes* (*Ochlerotatus*) *pionips* Dyar, 1919 (Diptera, Culicidae) in Germany. Check List..

[4] Kampen H., Walther D., Benelli G., Mehlhorn H. (2018). Vector potential of mosquito species (Diptera: Culicidae) occurring in Central Europe. Parasitology Research Monographs: Mosquito-Borne Diseases.

[5] Suk J. (2017). Preparedness for mosquito-borne diseases in Europe: The ECDC perspective. Eur. J. Public. Health.

[6] ECDC (European Centre for Disease Prevention and Control) (2023). Autochthonous Transmission of Chikungunya Virus in Mainland EU/EEA, 2007-Present. ECDC, Stockholm. https://www.ecdc.europa.eu/en/infectious-disease-topics/z-disease-list/chikungunya-virus-disease/surveillance-threats-and.

[7] ECDC (European Centre for Disease Prevention and Control) (2023). Autochthonous Vectorial Transmission of Dengue Virus in Mainland EU/EEA, 2010-Present. ECDC, Stockholm. https://www.ecdc.europa.eu/en/all-topics-z/dengue/surveillance-and-disease-data/autochthonous-transmission-dengue-virus-eueea.

[8] Sousa C.A., Clairouin M., Seixas G., Viveiros B., Novo M.T., Silva A.C., Escoval M.T., Economopoulou A. (2012). Ongoing outbreak of dengue type 1 in the Autonomous Region of Madeira, Portugal: Preliminary report. Eurosurveillance.

[9] Hernández-Triana L.M., Jeffries C.L., Mansfield K.L., Carnell G., Fooks A.R., Johnson N. (2014). Emergence of West Nile virus lineage 2 in Europe: A review on the introduction and spread of a mosquito-borne disease. Front. Public. Health.

[10] Vidaña B., Busquets N., Napp S., Pérez-Ramírez E., Jiménez-Clavero M.A., Johnson N. (2020). The role of birds of prey in West Nile virus epidemiology. Vaccines.

[11] Giesen C., Herrador Z., Fernandez-Martinez B., Figuerola J., Gangoso L., Vazquez A., Gómez-Barroso D. (2023). A systematic review of environmental factors related to WNV circulation in European and Mediterranean countries. One Health.

[12] Camp J.V., Nowotny N. (2020). The knowns and unknowns of West Nile virus in Europe: What did we learn from the 2018 outbreak?. Expert Rev. Anti Infect. Ther..

[13] Ziegler U., Lühken R., Keller M., Cadar D., van der Grinten E., Michel F., Albrecht K., Eiden M., Rinder M., Lachmann L. (2019). West Nile virus epizootic in Germany, 2018. Antivir. Res..

[14] Kampen H., Werner D., Mehlhorn H. (2011). Arthropod vectors and their growing importance in Europe. Parasitology Research Monographs: Progress in Parasitology.

[15] Pilaski J., Mackenstein H. (1985). Isolation of Tahyna virus from mosquitoes in two different European natural foci. ZBL Bakt. P..

[16] Jöst H., Bialonski A., Storch V., Günther S., Becker N., Schmidt-Chanasit J. (2010). Isolation and phylogenetic analysis of Sindbis viruses from mosquitoes in Germany. J. Clin. Microbiol..

[17] Scheuch D.E., Schäfer M., Eiden M., Heym E.C., Ziegler U., Walther D., Schmidt-Chanasit J., Keller M., Groschup M.H., Kampen H. (2018). Detection of Usutu, Sindbis, and Batai viruses in mosquitoes (Diptera: Culicidae) collected in Germany, 2011–2016. Viruses.

[18] Heym E.C., Kampen H., Krone O., Schäfer M., Werner D. (2019). Molecular detection of vector-borne pathogens from mosquitoes collected in two zoological gardens in Germany. Parasitol. Res..

[19] Jöst H., Bialonski A., Maus D., Sambri V., Eiden M., Groschup M.H., Günther S., Becker N., Schmidt-Chanasit J. (2011). Isolation of Usutu virus in Germany. Am. J. Trop. Med. Hyg..

[20] Kampen H., Holicki C.M., Ziegler U., Groschup M.H., Tews B.A., Werner D. (2020). West Nile virus mosquito vectors (Diptera: Culicidae) in Germany. Viruses.

[21] Kampen H., Tews B.A., Werner D. (2021). First evidence of West Nile virus overwintering in mosquitoes in Germany. Viruses.

[22] Hubálek Z. (2008). Mosquito-borne viruses in Europe. Parasitol. Res..

[23] Adouchief S., Smura T., Sane J., Vapalahti O., Kurkela S. (2016). Sindbis virus as a human pathogen—Epidemiology, clinical picture and pathogenesis. Rev. Med. Virol..

[24] Skogh M., Espmark Å. (1982). Ockelbo disease: Epidemic arthritis-exanthema syndrome in Sweden caused by Sindbis-virus like agent. Lancet.

[25] Brummer-Korvenkontio M., Vapalahti O., Kuusisto P., Saikku P., Manni T., Koskela P., Nygren T., Brummer-Korvenkontio H., Vaheri A. (2002). Epidemiology of Sindbis virus infections in Finland 1981–96: Possible factors explaining a peculiar disease pattern. Epidemiol. Infect..

[26] Hubalek Z., Sebesta O., Pesko J., Betasova L., Blazejova H., Venclikova K., Rudolf I. (2014). Isolation of Tahyna virus (California encephalitis group) from *Anopheles hyrcanus* (Diptera, Culicidae), a mosquito species new to, and expanding in, Central Europe. J. Med. Entomol..

[27] Camp J.V., Haider R., Porea D., Oslobanu L.E., Forgách P., Nowotny N. (2018). Serological surveillance for Tahyna virus (California encephalitis orthobunyavirus, Peribunyaviridae) neutralizing antibodies in wild ungulates in Austria, Hungary and Romania. Zoonoses Public Health.

[28] Stevanovic V., Vilibic-Cavlek T., Savic V., Klobucar A., Kovac S., Posavec M.C., Petrinic S., Bogdanic M., Santini M., Tesic V. (2022). Surveillance of Tahyna orthobunyavirus in urban areas in Croatia—The “One Health” approach. Trop. Med. Infect. Dis..

[29] Vázquez A., Jiménez-Clavero M.A., Franco L., Donoso-Mantke O., Sambri V., Niedrig M., Zeller H., Tenorio A. (2011). Usutu virus—Potential risk of human disease in Europe. Eurosurveillance.

[30] Grottola A., Marcacci M., Tagliazucchi S., Gennari W., Di Gennaro A., Orsini M., Monaco F., Marchegiano P., Marini V., Meacci M. (2017). Usutu virus infection in humans: Retrospective analysis in the municipality of Modena, Italy. Clin. Microbiol. Infect..

[31] Weissenböck H., Kolodziejek J., Fragner K., Kuhn R., Pfeffer M., Nowotny N. (2003). Usutu virus activity in Austria, 2001–2002. Microbes Infect..

[32] Becker N., Jöst H., Ziegler U., Eiden M., Höper D., Emmerich P., Schmidt-Chanasit J. (2012). Epizootic emergence of Usutu virus in wild and captive birds in Germany. PLoS ONE.

[33] Cadar D., Bosch S., Jöst H., Börstler J., Garigliany M.M., Becker N., Schmidt-Chanasit J. (2015). Putative lineage of novel African Usutu virus, central Europe. Emerg. Infect. Dis..

[34] Ziegler U., Jöst H., Müller K., Fischer D., Rinder M., Tietze D.T., Danner K.J., Becker N., Skuballa J., Hamann H.P. (2015). Epidemic spread of Usutu virus in southwest Germany in 2011 to 2013 and monitoring of wild birds for Usutu and West Nile viruses. Vector Borne Zoonotic Dis..

[35] Ziegler U., Fast C., Eiden M., Bock S., Schulze C., Hoeper D., Ochs A., Schlieben P., Keller M., Zielke D.E. (2016). Evidence for an independent third Usutu virus introduction into Germany. Vet. Microbiol..

[36] Cadar D., Lühken R., van der Jeugd H., Garigliany M., Ziegler U., Keller M., Lahoreau J., Lachmann L., Becker N., Kik M. (2017). Widespread activity of multiple lineages of Usutu virus, western Europe, 2016. Eurosurveillance.

[37] Sieg M., Schmidt V., Ziegler U., Keller M., Höper D., Heenemann K., Vahlenkamp T.W. (2017). Outbreak and cocirculation of three different Usutu virus strains in eastern Germany. Vector Borne Zoonotic Dis..

[38] Michel F., Sieg M., Fischer D., Keller M., Eiden M., Reuschel M., Ziegler U. (2019). Evidence for West Nile virus and Usutu virus infections in wild and resident birds in Germany, 2017 and 2018. Viruses.

[39] Werner D., Kampen H., Gutiérrez-López R., Logan J.G., Martínez-de la Puente J. (2022). Zoos and wildlife parks: A laboratory for the study of mosquito-borne wildlife diseases. Ecology of Diseases Transmitted by Mosquitoes to Wildlife—Ecology and Control of Vector-Borne Diseases.

[40] Rossi S.L., Ross T.M., Evans J.D. (2010). West Nile virus. Clin. Lab. Med..

[41] Komar N., Langevin S., Hinten S., Nemeth N., Edwards E., Hettler D., Davis B., Bowen R., Bunning M. (2003). Experimental infection of North American birds with the New York 1999 strain of West Nile virus. Emerg. Infect. Dis..

[42] Pietsch C., Michalski D., Münch J., Petros S., Bergs S., Trawinski H., Lübbert C., Liebert U. (2020). Autochthonous West Nile virus infection outbreak in humans, Leipzig, Germany, August to September 2020. Eurosurveillance.

[43] Ziegler U., Bergmann F., Fischer D., Müller K., Holicki C.M., Sadeghi B., Sieg M., Keller M., Schwehn R., Reuschel M. (2022). Spread of West Nile virus and Usutu virus in the German bird population, 2019–2020. Microorganisms.

[44] Ziegler U., Santos P.D., Groschup M.H., Hattendorf C., Eiden M., Höper D., Eisermann P., Keller M., Michel F., Klopfleisch R. (2020). West Nile virus epidemic in Germany triggered by epizootic emergence, 2019. Viruses.

[45] Sauer F., Jaworski L., Lühken R., Kiel E. (2020). Impacts of sampling rhythm and exposition on the effectiveness of artificial resting shelters for mosquito collection in northern Germany. J. Vector. Ecol..

[46] Becker N., Petric D., Zgomba M., Boase C., Madon M., Dahl C., Kaiser A. (2020). Mosquitoes: Identification, Ecology and Control.

[47] Ries C., Sharav T., Tseren-Ochir E.O., Beer M., Hoffmann B. (2020). Putative novel serotypes 33 and 35 in clinically healthy small ruminants in Mongolia expand the group of atypical BTV. Viruses.

[48] Proft J., Maier W.A., Kampen H. (1999). Identification of six sibling species of the *Anopheles maculipennis* complex (Diptera: Culicidae) by a polymerase chain reaction assay. Parasitol. Res..

[49] Kronefeld M., Werner D., Kampen H. (2014). PCR identification and distribution of *Anopheles daciae* (Diptera, Culicidae) in Germany. Parasitol. Res..

[50] Rudolf M., Czajka C., Börstler J., Melaun C., Jöst H., Thien H., von Badusche M., Becker N., Schmidt-Chanasit J., Krüger A. (2013). First nationwide surveillance of *Culex pipiens* complex and *Culex torrentium* mosquitoes demonstrated the presence of *Culex pipiens* biotype *pipiens/molestus* hybrids in Germany. PLoS ONE.

[51] Folmer O., Black M., Hoeh W., Lutz R., Vrijenhoek R. (1994). DNA primers for amplification of mitochondrial cytochrome c oxidase subunit I from diverse metazoan invertebrates. Mol. Mar. Biol. Biotechnol..

[52] Hebert P.D.N., Cywinska A., Ball S.L., deWaard J.R. (2003). Biological identifications through DNA barcodes. Biol. Sci..

[53] Vina-Rodriguez A., Sachse K., Ziegler U., Chaintoutis S.C., Keller M., Groschup M.H., Eiden M. (2017). A novel pan-Flavivirus detection and identification assay based on RT-qPCR and microarray. BioMed. Res. Int..

[54] Lambert A.J., Lanciotti R.S. (2009). Consensus amplification and novel multiplex sequencing method for S segment species identification of 47 viruses of the *Orthobunyavirus, Phlebovirus*, and *Nairovirus* genera of the family Bunyaviridae. J. Clin. Microbiol..

[55] Eshoo M.W., Whitehouse C.A., Zoll S.T., Massire C., Pennella T.D., Blyn L.B., Sampath R., Hall T.A., Ecker J.A., Desai A. (2007). Direct broad-range detection of alphaviruses in mosquito extracts. Virology.

[56] Del Amo J., Sotelo E., Fernández-Pinero J., Gallardo C., Llorente F., Agüero M., Jiménez-Clavero M.A. (2013). A novel quantitative multiplex real-time RT-PCR for the simultaneous detection and differentiation of West Nile virus lineages 1 and 2, and of Usutu virus. J. Virol. Methods.

[57] CDC (Centers for Disease Control and Prevention) West Nile Virus-Resources. http://www.cdc.gov/westnile/resourcepages/mosqSurvSoft.html.

[58] Eiden M., Ziegler U., Keller M., Müller K., Granzow H., Jöst H., Schmidt-Chanasit J., Groschup M.H. (2014). Isolation of Sindbis virus from a hooded crow in Germany. Vector Borne Zoonotic Dis..

[59] Ziegler U., Fischer D., Eiden M., Reuschel M., Müller K., Schwehn R., Schmidt V., Groschup M.H., Keller M. (2019). Sindbis virus—A wild bird associated zoonotic arbovirus circulates in Germany. Vet. Microbiol..

[60] Jansen S., Lühken R., Helms M., Pluskota B., Pfitzner W.P., Oerther S., Becker N., Schmidt-Chanasit J., Heitmann A. (2022). Vector competence of mosquitoes from Germany for Sindbis virus. Viruses.

[61] Modlmaier M., Kuhn R., Kaaden O.R., Pfeffer M. (2002). Transmission studies of a European Sindbis virus in the floodwater mosquito *Aedes vexans* (Diptera: Culicidae). Int. J. Med. Microbiol..

[62] Bateman A.C., Mueller S., Guenther K., Shult P. (2021). Assessing the dilution effect of specimen pooling on the sensitivity of SARS-CoV-2 PCR tests. J. Med. Virol..

[63] Bergman A., Dahl E., Lundkvist Å., Hesson J.C. (2020). Sindbis virus infection in non-blood-fed hibernating *Culex pipiens* mosquitoes in Sweden. Viruses.

[64] Hesson J.C., Verner-Carlsson J., Larsson A., Ahmed R., Lundkvist Å., Lundström J.O. (2015). *Culex torrentium* mosquito role as major enzootic vector defined by rate of Sindbis virus infection, Sweden, 2009. Emerg. Infect. Dis..

[65] Holicki C.M., Scheuch D.E., Ziegler U., Lettow J., Kampen H., Werner D., Groschup M.H. (2020). German *Culex pipiens* biotype *molestus* and *Culex torrentium* are vector-competent for Usutu virus. Parasit. Vectors.

[66] Körsten C., Al-Hosary A.A., Holicki C.M., Schäfer M., Tews B.A., Vasić A., Ziegler U., Groschup M.H., Silaghi C. (2023). Simultaneous coinfections with West Nile virus and Usutu virus in *Culex pipiens* and *Aedes vexans* mosquitoes. Transbound. Emerg. Dis..

[67] Frank C., Offergeld R., Lachmann R., Stark K., Schmidt-Chanasit J. (2023). Saison stechmückenübertragener Krankheitserreger beginnt. Epidemiol. Bull..

[68] Jaworski L., Sauer F., Jansen S., Tannich E., Schmidt-Chanasit J., Kiel E., Lühken R. (2022). Artificial resting sites: An alternative sampling method for adult mosquitoes. Med. Vet. Entomol..

