# Supplementary material for: Viral RNA in Mosquitoes (Diptera: Culicidae) Collected between 2019 and 2021 in Germany
Source: Viruses. 2023 Nov 23;15(12):2298. doi: 10.3390/v15122298 (PMC10746995; doi:10.3390/v15122298)
Supplement: Supplementary file 1 [file viruses-15-02298-s001.zip › Rau-Köchling_Supplementary Material.pdf]

Supplementary Material

Table S1: Virus detections in mosquitoes from Germany in 2019 to 2021.

Virus	Location	Trapping site coordinates	Trap type	Mosquito taxon	Collection date	No. mosquitoes per sample/pool
Sindbis virus	Berlin	52.506555<br>13.532151	BG-Sentinel	*Cx. torrentium*	17 Apr 2019	1
	Berlin	52.506555<br>13.532151	BG-Sentinel	*Cx. pipiens*<br>biotype *pipiens*	05 Jun 2019	1
	Berlin	52.506555<br>13.532151	BG-Sentinel	*An. maculipennis*	09 Jul 2019	1
	Beerfelde	52.434789<br>14.041937	BG-Sentinel	*Cx. pipiens*<br>biotype *pipiens*	29 Aug 2019	1
	Groß Kreutz	52.396340<br>12.764405	BG-Sentinel	*An. messeae*	03 Sept 2019	1
	Dannenreich	52.315134<br>13.748239	BG-sentinel	*Cx. pipiens*<br>biotype *pipiens*	20 Oct 2019	1
	Kunsterspring	53.029358<br>12.767277	BG-Sentinel	*Ae. annulipes*<br>group	21 Mai 2020	1
	Moos	48.775120<br>12.957347	Popup bag	*Ae. sticticus*	24 Jun 2020	5
	Eggenstein-<br>Leopoldshafen	49.101898<br>8.382824	BG-Sentinel	*An. daciae*	06 Jul 2020	1
	Moos	48.775120<br>12.957347	Popup bag	*Ae. sticticus*	08 Jul-2020	1
	Berlin	52.497580<br>13.532430	BG-Sentinel	*Cx. pipiens*<br>biotype *pipiens*	02 Sept 2020	1
	Kunsterspring	53.029358<br>12.767277	BG-Sentinel	*An. claviger*	17 Jun 2021	1

	Eggenstein-Leopoldshafen	49.101824 8.382647	BG-Sentinel	*Ae. cinereus/geminus*	20 Jun 2021	10
	Goldenstedt	52.727667 8.391128	BG-Sentinel	*Cx. pipiens* biotype *pipiens*	28 Jun 2021	6
	Aken	51.855160 12.034553	EVS trap	*Cx. modestus*	07 Jul 2021	10
	Gera	50.865227 12.054065	BG-Sentinel	*Cx. pipiens* biotype *pipiens*	02 Aug 2021	1
	Irgenöd	48.563304 13.251970	BG-Sentinel	*Cx. pipiens* complex	26 Aug 2021	6
	Eggenstein-Leopoldshafen	49.101824 8.382647	BG-Sentinel	*Ae. sticticus*	28 Aug 2021	10
	Neustrelitz	53.357094 13.067817	BG-Sentinel	*Ae. vexans*	05 Sept 2021	5
	Goldenstedt	52.727667 8.391128	BG-Sentinel	*Cs. morsitans/fumipennis*	06 Sept 2021	2
	Bernburg	51.793968 11.728581	Aspirator	*Cx. pipiens* complex	10 Nov 2021	10
Usutu virus	Groß Kreutz	52.396340 12.764405	BG-Sentinel	*Cx. pipiens* biotypes *pipiens* and *molestus* or hybrids	03 Sept 2019	16
	Schorfheide	52.920387 13.556368	BG-Sentinel	*Ae. vexans*	01 Jul 2020	1
	Berlin	52.497580 13.532430	BG-Sentinel	*Cx. pipiens* biotypes *pipiens* and *molestus* or hybrids	12 Aug 2020	7
	Dresden	51.036972 13.753514	BG-Sentinel	*Cx. pipiens* biotype *pipiens*	30 Sept 2020	1

	Angermünde	53.007160 13.990036	BG-Sentinel	*Ae. vexans*	03 Jun 2021	1
	Berlin	52.497580 13.532430	BG-Sentinel	*Cx. pipiens* biotype *pipien*s and *Cx. torrentium*	02 Aug 2021	10
West Nile virus	Magdeburg	52.167825 11.644682	BG-Sentinel	*Cx. pipiens* biotype *pipiens*	27 Jun 2020	1
	Bernburg	51.036972 13.753514	BG-Sentinel	*Cx. pipiens* biotype *pipiens*	20 Aug 2020	1
	Dresden	51.036972 13.753514	BG-Sentinel	*Cx. pipiens* biotype *pipiens*	02 Sept 2020	1
	Magdeburg	52.167825 11.644682	BG-Sentinel	*Cx. pipiens* biotype *pipien*s and *Cx. torrentium*	12 Jun 2021	10
	Magdeburg	52.167825 11.644682	BG-Sentinel	*Cx. pipiens* biotype *pipiens*	25 Jun 2021	1
	Magdeburg	52.167825 11.644682	BG-Sentinel	*Cx. pipiens* biotype *pipiens*	06 Jul 2021	10
	Wittenberg	51.869111 12.641278	BG-Sentinel	*Cx. pipiens* biotype *pipiens*	18 Jul 2021	10
	Magdeburg	52.167825 11.644682	BG-Sentinel	*Cx. pipiens* biotypes *pipiens* and *molestus* or hybrids	01 Aug 2021	10
	Magdeburg	52.167825 11.644682	BG-Sentinel	*Cx. pipiens* biotype *pipien*s and *Cx. torrentium*	01 Aug 2021	10

Magdeburg	52.167825 11.644682	BG-Sentinel	*Cx. pipiens* biotype *pipien*s and *Cx. torrentium*	01 Aug 2021	10
Magdeburg	52.167825 11.644682	BG-Sentinel	*Cx. pipiens* biotype *pipien*s and *Cx. torrentium*	01 Aug 2021	10
